# Barriers and facilitators to healthcare access for homeless people in four European countries

**DOI:** 10.1093/eurpub/ckac129.068

**Published:** 2022-10-25

**Authors:** T Schiffler, C Carmichael, L Lehner, A Gil-Salmeron, M Kouvari, P Karnaki, I Grabovac

**Affiliations:** 1 Center for Public Health, Department of Social and Preventive Medicine, Medical University of Vienna, Vienna, Austria; 2 Centre for Health, Performance, Wellbeing, Anglia Ruskin University, Cambridge, UK; 3 Ambermed, Diakonie Refugee Services, Vienna, Austria; 4 International Foundation for Integrated Care, Oxford, UK; 5 PROLEPSIS Institute, Athens, Greece

## Abstract

**Background:**

People experiencing homelessness (PEH) have higher prevalence of adverse health outcomes and premature mortality compared to the general population, and often experience significant barriers in accessing healthcare services. This study aimed to better understand the health needs of PEH, as well as to identify the barriers and facilitators to their timely and equitable access to health services from the perspective of PEH and relevant health professionals and social care workers.

**Methods:**

During autumn 2021, a cross-national qualitative study was conducted within the framework of the Horizon 2020 funded CANCERLESS project. Semi-structured interviews were conducted across four European settings (Austria, Greece, Spain and the UK). Interviews were audio-recorded, transcribed verbatim and analyzed according to the inductive thematic approach set out by Saldaña (2021).

**Results:**

In total, 69 interviews were completed with a sample comprising 15 professionals working in homelessness support services, 19 health professionals, and 35 PEH. Three overarching themes relating to the research question were identified: (a) Health needs of people experiencing homelessness; (b) Barriers to access healthcare services and (c) Facilitators to access healthcare services. Overall, the general health of PEH was depicted as extremely poor and mainstream health services were portrayed as ill-equipped to respond to the needs of this population, with many organizational and system-level barriers noted. Tailored approaches to care, and in particular involving trusted professionals in the delivery of care, were identified as a key strategy for overcoming existing barriers.

**Conclusions:**

While a number of context-specific findings were identified, results indicated there to be a high degree of overlap and consistency in the health needs of PEH, and in the barriers and facilitators that exist when accessing healthcare across four different healthcare systems.

**Key messages:**

• Homelessness is a determinant of health that is linked to poor health outcomes. Tailored approaches that draw upon trusting relationships have the potential to overcome this problem.

• The array of identified barriers indicates that general healthcare services are not currently structured in a way that facilitates timely and equitable access and appropriate care for PEH.

